# A report on the performance of the Gilford EIA 50 automated micro-ELISA processor

**DOI:** 10.1155/S1463924682000339

**Published:** 1982

**Authors:** R. Goodburn, D. L. Williams, V. Marks

**Affiliations:** 1 Department of Clinical Biochemistry Ashford Hospital London Road Ashford, Middlesex UK; 2 Department of Biochemistry University of Surrey Surrey Guildford UK


Journal of Automatic Chemistry, Volume 4, Number 3 (July–September 1982), pages 129–134

### A report on the performance of the Gilford EIA 50 automated micro-ELISA processor

**R. Goodburn, D. L. Williams**

*Department of Clinical Biochemistry, Ashford Hospital, London Road, Ashford, Middlesex, UK*

**and V. Marks**

*Department of Biochemistry, University of Surrey, Guildford, Surrey, UK*

### Introduction

The enzyme-linked immunosorbent assay (ELISA) technique is widely used for the qualitative detection of antigens and antibodies [1], and results are generally assessed subjectively. For the more recently introduced quantitative methods the end-point is measured colorimetrically or fluorimetrically.

Since the introduction of ELISA methodologies, equipment has been produced to cope with most of the pipetting and washing procedures. Apparatus is also available for colorimetric assessment of the end-point. Most of these pieces of equipment will perform only one of these tasks, and in order to automate the entire ELISA procedure, three or four pieces of expensive equipment were until quite recently necessary.

Gilford Instruments (Corning Medical and Scientific, Corning Ltd, Halstead, Essex, UK) have now produced a complete ELISA system, the EIA 50. The EIA 50 uses specially produced polystyrene cuvettes, which are manufactured in ranks of 10, allowing the assay of relatively small batches of samples. The ranks of cuvettes fit in batches of five into moulded plastic bases, which can be clipped together to allow the processing of as many cuvettes as is practicable in the assay. The entire ELISA procedure is performed in the cuvettes, and the final colour is read through the lower part of the cuvettes, which have optically clear walls.

The processor consists of a cuvette transport system, colorimeter. wash/dry system, dispenser and printer. The various functions are microprocessor controlled, allowing eight separate procedures to be performed, with a range of reagent volumes, wash conditions and cycle times.

It should be mentioned that a diluter is not incorporated into the machine. Therefore each serum sample under investigation must be diluted either manually, or with a diluter, and then added to the appropriate cuvette manually. It is difficult to visualize a system which would allow these steps to be automated. The procedures (programs) selectable by the operator are shown in table 1.

The system was evaluated in the authors' laboratory with particular reference to auto-antibody studies [2, 3]. However, the evaluation procedures used are not particularly specific to this application, and micro-ELISA methods for antibodies to infectious diseases are likely to give similar results. Wherever applicable, the evaluation was performed according to the recommendations of Broughton *et al.* [4].

The basic indirect, or sandwich, micro-ELISA procedure used by the authors consists of the following steps:

(1) Adsorption of antigen onto the solid phase by incubation in coating buffer at 37°C for 2 h.
(2) Washing the solid phase to remove antigen not adsorbed.
(3) Addition of serum containing antibodies specific for the adsorbed antigen, followed by incubation for 2 h at 37°C.
(4) Washing to remove unabsorbed antibody.
(5) Addition of the anti-Ig/enzyme conjugate with a further 2 h incubation at 37°C.
(6) Washing, followed by addition of substrate, and after colour generation, addition of stop reagent and measurement of absorbance.

### Materials

### *Micro-ELISA materials*

Throughout this study goat anti-human immuno-globulins (Ig) horse-radish-peroxidase was used as the labelled antibody. This was prepared by the two-step glutaraldehyde method [5].

Detection of bound peroxidase activity was by means of the o-phenylene diamine/hydrogen peroxide reaction.

### *Buffers*

(1) Coating buffer: carbonate/bicarbonate buffer, pH 9·6, 1·59 g $Na_2CO_3$, 2·93 g Na $HCO_3$ made up to 1 l with distilled water. This buffer was prepared freshly every week.
(2) Wash buffer; PBS/Tween/gelatin, pH 7·4, 40 g NaCl,

*Table 1. Programs available on the Gilford EIA 50 ELISA processor.*

*Modes of operation*

| *Setting* | *Operation* |
|---|---|
| 0 (Blank 1) | Manual read of wells (Test mode) |
| 0 (Blank 2) | Flush; enter diagnostics |
| 1 | Aspirate: wash: (*N* cycles—*N* soak s): (filled): rack |
| 2 | Aspirate: wash: (*N* cycles—*N* soak s): (empty): rack |
| 3 | Aspirate: wash: (*N* cycles—*N* soak s): (empty): dispense: rack |
| 4 | Aspirate: wash: (*N* cycles—*N* soak s): (empty): dispense: read: rack |
| 5 | Dispense: rack |
| 6 | Dispense: read: rack |
| 7 | Read: rack |
| 8 | Fast read 80 s |
| 9 | RS232 interface |

0142 0453/82/0403 0129 $05·00 © 1982 Taylor & Francis Ltd

1 g $KH_2PO_4$, 14·5 g $Na_2HPO_4$. $12H_2O$ 2·5 g gelatine and 2·5 ml Tween 20 made up to 5 l with distilled water.

(3) Substrate buffer; citrate/phosphate buffer, pH 5·0, 24·3 ml 0·1 M citric acid, 25·7 ml 0·2 M $Na_2HPO_4$ made up to 1 l with distilled water.

### *Substrate*

20 mg o-phenylene diamine was dissolved in 50 mls citrate/phosphate buffer, and 20 $\mu$l of hydrogen peroxide were added. This solution was prepared immediately before use.

### *Other materials*

In studies on the precision of the dispenser, $^{125}$I-labelled thyroxine was used diluted in the buffer appropriate for the particular part of the assay under scrutiny. This compound was chosen since it was pertinent to the study for which the instrument was being used.

In experiments involving human IgG, serum samples with normal concentrations of total proteins and albumin were used.

### Method

The following features of the system were investigated: Literature; Presentation of equipment; Electrical and mechanical safety; Dispenser; Photometer and filter; Cuvette washing system; Cuvettes; General aspects.

### *Literature*

A fully descriptive operator's manual is supplied with the analyser. The manual gives details of a preventative maintenance schedule and has a comprehensive system of safety cautions and warnings for the operator.

With the machine used in this investigation, a service manual was also supplied which gives more detailed maintenance, service and repair information.

The operator's manual has been necessary for such operations as syringe servicing, lamp alignment and photometer calibration. The service manual, although potentially useful, is intended for use by trained service personnel, and it should be noted that any applicable guarantee may be prejudiced by untrained staff undertaking some of the procedures. However, the service manual does have a section on failure analysis which gives considerably more information on this topic than does the operator's manual, and this has been found to be useful.

### *Presentation of equipment*

The machine which was evaluated was an ex-demonstration model which, before delivery, had been fully reconditioned and serviced to the standard of a new machine. So it is not possible to comment on the presentation and installation of a new instrument.

In general, the operations of the machine are easily performed, all programming being carried out by means of four sets of thumb-wheels, three push-buttons and two knobs.

In order to load the required program into the memory, the appropriate number is selected with a thumb-wheel, and the 'start' button is pressed. The cassette (the plastic base with up to five ranks of cuvettes) is then placed in position and the start button pressed again to run the program. In some circumstances it is difficult to know whether the program has been loaded and is ready to run, or whether the previous program is still loaded. An additional 'load' button could overcome this potential problem.

### *Electrical and mechanical safety*

The equipment was checked by the authors' electro-mechanical equipment department for electrical safety, and considered satisfactory. However, it was often necessary to operate the machine with the main cover removed thus creating the possibility of accidental spillage into the compartment housing the electronic equipment.

The only potential mechanical hazard identified was the possibility of fingers becoming trapped between the piston and end-stop of the dispenser. This did not actually happen during the evaluation.

### *Dispenser*

The dispenser incorporated in the analyser is Gilford Instruments' automatic dispenser fitted with a 1 ml Hamilton syringe and remotely controlled by the central microprocessor. The required volumes are selected and set from a turret of steel rods which, when placed under the syringe plunger, prevents its progress beyond a certain point, thus dictating the volume dispensed. Small-bore tubing is used to keep dead volumes to a minimum, and priming, by means of a toggle switch, is fast.

The precision, accuracy, and general performance, of the dispenser were checked as follows:

(1) *Precision*. This was tested at the 250 $\mu$l (25% of full stroke) and 50 $\mu$l (5% of full stroke) levels. $^{125}$I-Thyroxine was diluted in wash buffer to a concentration which gave approximately 30 000 counts/min. at the volume setting used. Thirty dispensings were made, using the remote control of the microprocessor, but with the dispensed liquid being directed into polystyrene test-tubes. The precisions at the 250 $\mu$l and 50 $\mu$l levels were 1·26% and 2·39% respectively (coefficients of variation), after correction for counting errors. The manufacturer states that the precision at full stroke, using a 2·5 ml syringe, is 0·05% (CV).
(2) *Accuracy*. The dispenser syringe and tubing were thoroughly cleaned, and primed with deionized water. Forty 250 $\mu$l and fifty 50 $\mu$l aliquots were then dispensed into two pre-weighed plastic containers. The volumes dispensed as measured gravimetrically were 253 $\mu$l and 51 $\mu$l.
(3) *General performance*. A recurring problem was encountered with the dispenser in the early stages of this study. The syringe piston is driven by a servo motor via a continuous loop of nylon cord. Occasionally this slipped on one of the pulleys preventing the switching of a microswitch. When this fault occurred, unless the operator was immediately available to dismantle the dispenser, the timing sequence for a particular group of cuvettes was invariably lost. The fault was remedied by tightening the cord and adjusting the switching mechanism. These adjustments were made by a service engineer and the problem has not recurred in the two months since the work was carried out.

### *Photometer and filter*

The photometer is of the Silicon photo-diode type, with a range from 0 to 2 absorbance units.

To check the precision of reading, two cuvettes in one rank were used. The enzyme-catalysed reaction was performed and stopped when the absorbance had reached approximately 1·0. The absorbaces were then read using the 'fast read' program. Ten readings for each cuvette were taken and the mean ± standard deviation calculated for each set of readings. The results for each cuvette were 1·155±0·0016 (CV 0·14%) and 1·153±0·0018 (CV 0·16%) which were considered to be acceptable.

Two interference filters (405 nm and 492 nm) were supplied with the machine. In this study, only the 492 nm filter was used; this gave a transmission peak at 492 nm with a narrow band width. The filters are easily exchangeable on the instrument. A tungsten lamp is used and the system is blanked by adjusting the current to the lamp. The stability of the photometer system is stated by the manufacturer to be less than 1%/h. The authors found, after an initial warm-up period of 50 min., that there was no instability over a 2 h period at the blank level (i.e. absorbance = 0·00).

### *Cuvette washing system*

During the micro-ELISA method, it is necessary to wash the cuvettes after each incubation and before the addition of the next reagent. It is essential that this washing is efficient.

The cuvettes are washed in ranks of 10. The reagent in the cuvettes is aspirated, by vacuum, through metal tubes which reach almost to the bottom of each cuvette. 750 $\mu$l of wash buffer is then dispensed into each cuvette, and this is aspirated after a pre-programmed soak time. A further wash cycle may then begin, reagent may be dispensed into the cuvettes, or the cuvettes transferred back into the cassette. One to nine wash cycles may be carried out with a soak time of 0–99 s for each cycle.

The vacuum pump supplied with the instrument, also a refurbished 'as new' ex-demonstration unit, broke down after four months of reasonably heavy use; it was exchanged by a service engineer within 48 h and the replacement unit has given satisfactory service for four months.

The precision of the addition of wash buffer was not assessed, as the volume (750 $\mu$l) dispensed was considered to be more than adequate.

The optimum washing conditions used in the micro-ELISA methods were established as follows. A typical antibody-antigen reaction was carried out by incubating, for 2 h at 37°C, cuvettes in which human IgG had been adsorbed, with anti-human IgG/peroxidase at the appropriate dilution. Each rank of 10 cuvettes was then subjected to one of a variety of washing procedures, ranging from one to eight washes and a soak time of 0, 5 or 10 s. Subsequently the enzyme reaction was performed, and the absorbance measured. Timing of the enzyme reaction was strictly maintained. The results in figure 1 show that six washes are satisfactory at all soak times. In these assays a 5 s soak time was adopted. This washing procedure was found to be satisfactory, and there is sufficient spare capacity to enable the assumption that wash procedures for other ELISA assays can be adequately achieved. Two drawbacks of the wash system were noted:

(1) In setting up the machine for an assay, the wash system peristaltic pump tubes must initially be placed in position and then primed with wash buffer. Positioning the pump tubes should be a relatively simple procedure but on our machine the moveable block holding the tubes in position was occasionally incorrectly positioned because of difficulty of access to the block. This may well lead to inconsistent wash procedures, with consequent loss of precision.

(2) Priming the pump tubes is somewhat cumbersome, involving the use of one of the programs, together with a special button to give continuous operation of the peristaltic pump. Wash buffers that contain detergents and/or proteins have a tendency to foam, and this prevents efficient fast priming of the tubes. Experience has shown that extensive priming (up to 2 min.) may be necessary, with a consequent loss of a significant volume of buffer.

### *Cuvettes*

During the progress of this study, three batches of cuvettes were supplied. The first batch used were shown to suffer from an 'edge' or 'smile' effect, which resulted in absorbances from the cuvettes at the ends of each rank being considerably higher than those from the middle cuvettes. This effect is caused by inconsistent inter-cuvette antigen-binding characteristics. The second batch of cuvettes were shown to be relatively free from this defect, with only occasional marginally raised absorbances on cuvettes 1 and 10 in a rank. The third batch of cuvettes obtained showed an edge effect which, although not as marked as that seen with the first batch, has nevertheless caused some problems. The edge effects of the three batches of cuvettes are demonstrated in figure 2.

Recently the manufacturer has informed us that a major contributing factor to the edge effect is high incubation temperatures (for example 37°C). They have suggested that the use of room-temperature incubation reduces the effect.

The plastic bases of the cassettes have been modified, by the addition of hooks, allowing chaining of the cassettes. This then allows continuous processing of large numbers of cuvettes if necessary. It should be mentioned, however, that there is an inherent drawback to this system. Because of the number of washes required for each rank of cuvettes, with a soak time generally of 5 or 10 s, it may take up to 10 min. to process each cassette of five cuvette ranks. So if the colour reaction is particularly rapid it may not be possible to stop the reaction in sufficient time to prevent the absorbances becoming too high for measurement. Thus it is important either to process small enough batches of cuvettes to allow the measurement of the final absorbance with a relatively fast enzyme reaction, or to choose assay conditions which keep the enzyme reaction slow. (Alternatively, the final colour may be diluted for reading.)

In order to keep assay conditions as constant as possible, it is advisable to coat as many cuvettes as possible with antigen or antibody prior to commencing a given study. This obviously, therefore, requires the easy availability of cuvettes in large numbers. Unfortunately, experience has shown that the instrument supplier has not been able to supply the cassettes in quantities greater than 10 (i.e. 500 cuvettes) at any given time. This has led to considerable problems in the studies being undertaken, and must be considered to be a serious drawback in the use of the equipment, especially since at present there are no other suppliers of the cuvettes.

It should also be noted that the cuvettes are disposable, and in the authors' laboratory have not been reusable, even after extensive detergent washing.

### *General aspects*

### *Cycle time*

A cycle time thumb-wheel is provided. This allows accurate rank-to-rank timing of all stages in the ELISA procedure; it is especially important to maintain accurate timing of the enzyme reaction throughout the whole batch of samples. The timing of each procedure from cuvette to cuvette within each rank is

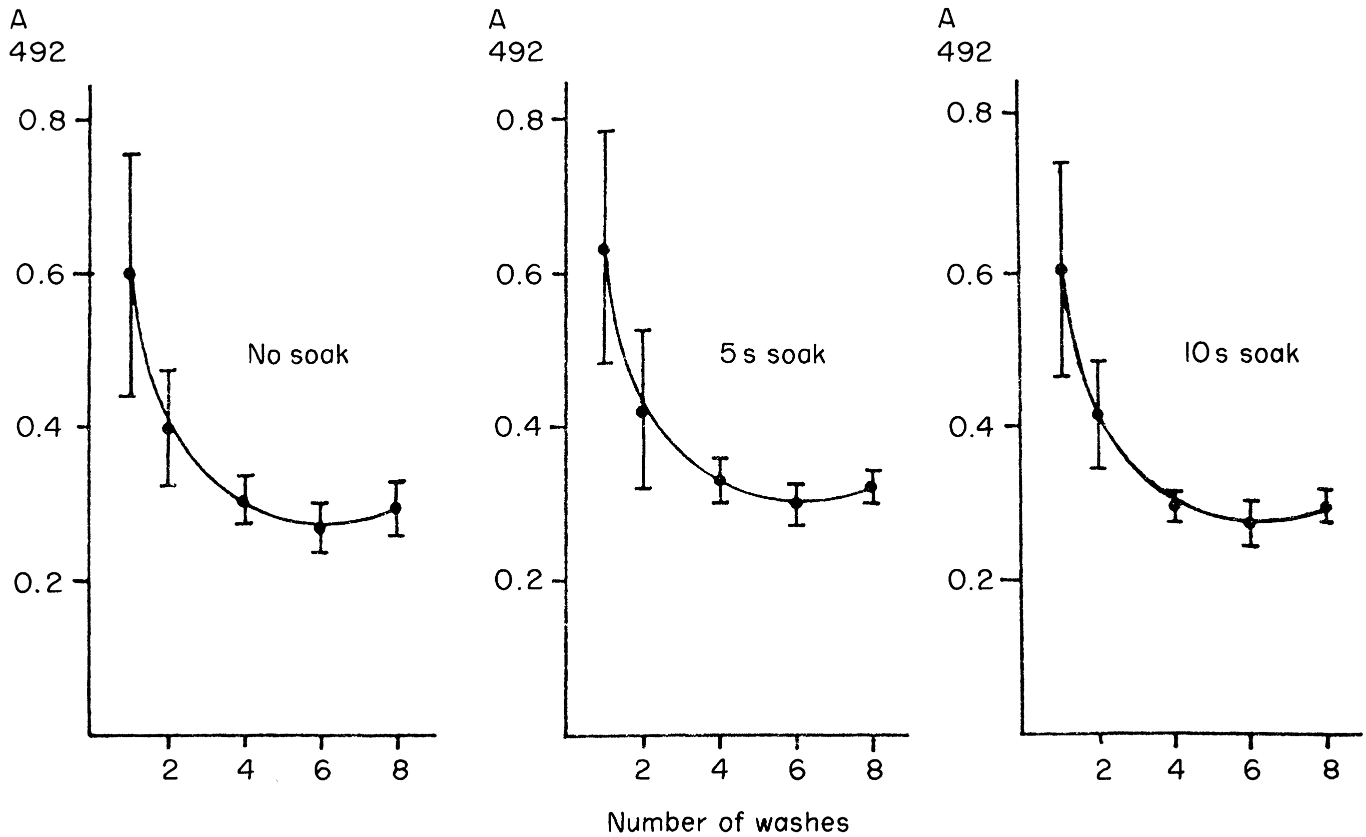

*Figure 1. Effect of soak time and number of washes on final ELISA absorbance using the Gilford EIA 50 processor. The points represent means (together with standard deviations) of results from 10 cuvettes.*

automatically maintained by microprocessor control of each program.

*Carry-over*

The only operation in which carry-over is a potential problem is washing the cuvettes. However, in the wash mechanism, both the wash solution dispensing tube and the aspiration tube are washed in each cycle and if more than one wash cycle is used no carry-over is evident. All other operations are localized in individual cuvettes and no cross-contamination occurs. It is important that the dispenser should be thoroughly flushed and washed between dispensing each reagent, especially after the antibody-enzyme conjugate and before the substrate. 0·1 M HCl is generally used to wash the dispenser briefly at this stage.

*Overall performance*

The overall performance of the system was compared with the classical manual micro-ELISA procedure using microtitre plates. Briefly, the procedure used consisted of:

(1) Adsorption of antigen onto either the microtitre plate wells or cuvettes.
(2) Washing. In the microtitre plate method this consisted of three washes, with a 3 min. soak. The cuvettes were washed six times, with a 5 s soak.
(3) Addition of serum containing antibodies against the particular antigen. In this case, antiserum was diluted to $10^{-3}$, $10^{-4}$, $10^{-5}$, $10^{-6}$ and $10^{-7}$ and 10 cuvettes and 10 wells were innoculated with each dilution.
(4) Following incubation, the plates and cuvettes were again washed as above.
(5) Addition of anti-IgG/peroxidase conjugate at an appropriate dilution.
(6) Incubation, followed by washing.
(7) Enzyme reaction. This was controlled in the case of the cuvettes by appropriate programming, and in the case of the microtitre plates by subjective timing. Absorbances in the microtitre plates method were measured in a modified Vitatron colorimeter. In the cuvette method the absorbances were measured on the EIA 50 using one of the programs.

The results of this comparison are shown in figure 3 and table 2. It may be seen from the figure that the antigen-binding capacity of the cuvettes is better than that of the microtitre plate. However, the precision obtained with the microtitre plates is slightly better than that of the cuvettes, as shown in the table.

### Discussion

The Gilford EIA 50 ELISA processor has been used in the authors' laboratory for studies on thyroid auto-antibodies. The machine has proved to be a useful tool for this purpose, showing some advantages over the previously used manual system (with microtitre plates).

The processor performs automatically and precisely most of the time-consuming and technically arduous procedures in the micro-ELISA method.

The machine has performed satisfactorily, with the exception of the dispenser, which initially caused occasional but serious problems. However, by far the most serious drawback of the system is the availability and quality of the cuvettes. It is unfortunate that the only UK supplier of the cuvettes for use in the machine was unable to supply the authors' requirements for

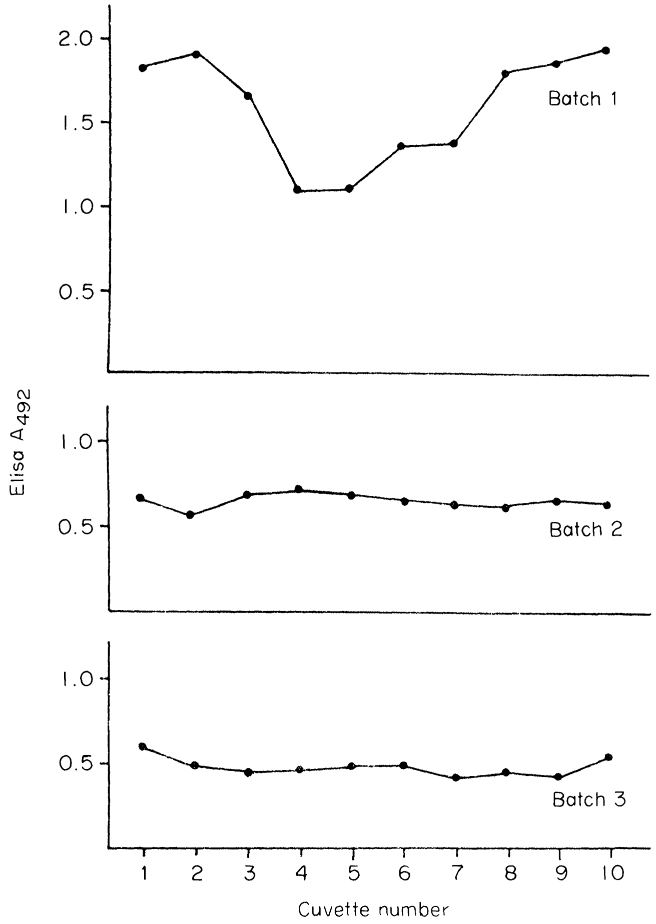

*Figure 2. Edge effect in three batches of cuvettes. The graphs shown are typical results obtained with ranks of cuvettes from each batch.*

a period of two months. Whilst assurances have been given that the problem of cuvette supplies have been resolved, there is no indication that the cuvette quality will be improved and standardized.

In the study currently being undertaken in this laboratory, it is essential that objective, precise and quantitative results should be obtained. However, in many ELISA procedures, a subjective qualitative or semi-quantitative result only is required. In these cases it is unlikely that the expense of automated processing equipment of this kind is justifiable. Kits are currently available which allow ELISA methods using precalibrated dropper bottles for reagent dispensing and sample dilution. In these tests the end-point is usually easily identifiable as positive or negative. Some gradation between these extremes is usually possible by subjective assessment.

Gilford Instruments (Corning Ltd) have produced an instrument which allows almost complete automation of the steps of the enzyme-linked immuno-sorbent assay. The advantages of one machine performing several procedures at a cost considerably lower than that of several machines performing

*Table 2. Comparison of precision of response obtained in ELISA procedure with EIA 50 cuvettes and microtitre plates.*

| Antiserum dilution | Coefficient of variation (CV%) Cuvettes | Microtitre plate |
|---|---|---|
| $10^{-3}$ | 3·1 | 1·7 |
| $10^{-4}$ | 2·3 | 1·4 |
| $10^{-5}$ | 3·8 | 3·2 |
| $10^{-6}$ | 8·4 | 6·2 |
| $10^{-7}$ | 8·9 | 8·8 |

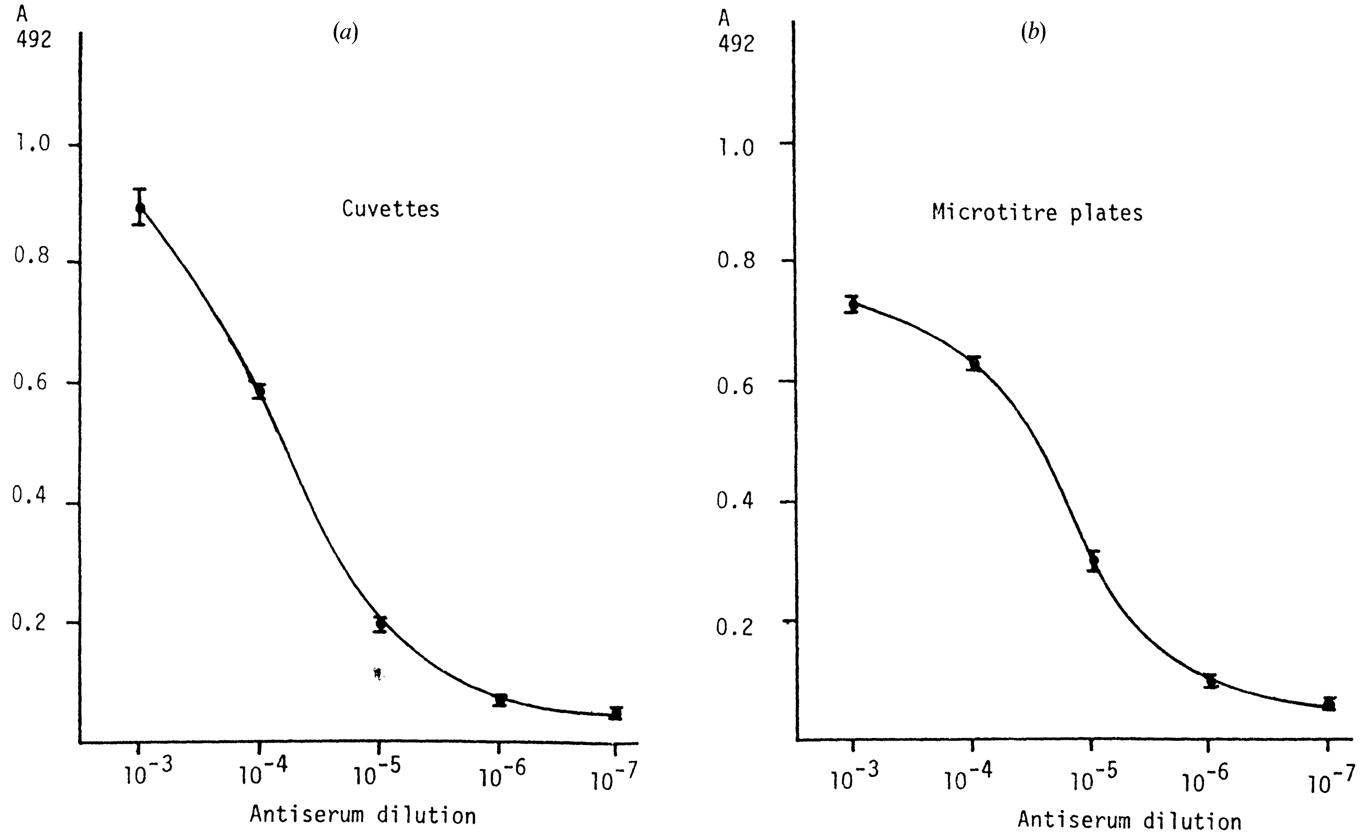

*Figure 3. Comparison of overall performance of Gilford EIA 50/cuvette system (a) with a manual/microtitre plate system (b). Points represent means (together with standard deviations) of results from 10 cuvettes or microtitre plate wells.*

individual steps are obvious. It remains to be seen whether the use of the cuvettes proves to be advantageous over the other adsorption media, in view of the wide availability of the latter.

### Acknowledgements

This work was supported by a grant made available under the locally organized research scheme of the South West Thames Regional Health Authority. Thanks are due to Mrs V. Stevenson for manuscript preparation.

### References

1. VOLLER, A., BIDWELL, D. E. and BARTLETT, A., *W.H.O. Bulletin*, **53** (1976), 55–65.
2. GOODBURN, R., WILLIAMS, D. L. and MARKS, V., *Journal of Clinical Pathology*, **34** (1981), 1026–1031.
3. GOODBURN, R., WILLIAMS, D. L. and MARKS, V., *Clinica Chimica Acta*, **119** (1982), 291–297.
4. BROUGHTON, P. M. G., GOWENLOCK, A. H., MCCORMACK, J. J. and NEILL, D. W., *Annals of Clinical Biochemistry*, **11** (1974), 207–218.
5. AVRAMEAS, S. and TERNYCK, T., *Immunochemistry*, **8** (1971), 1175–1179.

### 'Japanese Journal of Clinical Laboratory Automation'

*JJCLA published the following articles in its January 1982 issue (Vol. 7, No. 1):*

Report of interface committee: Recommended practice for analytical device and computer interfaces (*Kōki Motegi*).

*Educational lectures*

The present status and standardization of automatic instruments for blood coagulation testing in Japan (*Hirofumi Suzuki*).
The Automation of hospital bacteriology laboratories: present and future (*Akio Kobayashi*).
Clinical microbiology tests and automation (*Itaru Furuta*).

*Panel discussions*

Problems of management in hospital laboratories in the automated age (*Hiroshi Kiyose*).
From the viewpoint of applications of computers in the laboratory information system (*Seiji Kasuga*).
The ideal clinical laboratory in the automated generation (*Hideto Kushiro*).
Laboratory management in the future (*Takeyoshi Fujisawa*).
Problems of clinical laboratories in the time of automation: from the standpoint of the education of medical technologists (*Kihachirö Takahara*).

*Reports*

Evaluation of a total systematization of a haematological examination system (*Takuro Morii*).
The cell analyser MICROX system applied to automatic classification of degenerated white cells (*Hisatsugu Yamazaki*).
A study on the reliability of an automated blood cell differential analyser based on pattern recognition (*Teruo Yamada*).
Automated thromboplastin screening test using the Cobas Bio centrifugal analyser and chromogenic substrate (*Kiichi Asai*).
An automated method for the determination of plasma AT III with a chromogenic substrate using the Hitachi 706D (*Masayuki Soma*).
Development of an automatic method for determination of plasma antithrombin III using a chromogenic substrate (*Noriyo Hiraki*).
Use of sartorius cellulose acetate film on a fully automated electrophoresis analyser (AES) (*Atuko Kihara*).
About aspects of protein using the AES (electrophoresis autoanalyser) (*Yuriko Horikawa*).
Assessment of the Olympus AES 200 (*Eiji Katou*).
Evaluation of a new automated electrophoresis system (AES200)—with special reference to applications for urinary protein fractionation (*Junko Tanzawa*).
Development of cassette-type electrophoresis (*Hisatsugu Yamazaki*).
The evaluation of Jookoo (a cassette-type fully automatic electrophoresis system) CTE-500 (*Hisatsugu Yamazaki*).
Studies on the quality controls of automatic haematological and biochemical analysers (*Masatake Matsuda*).

*Original articles*

Determination of guanine deaminase activity in serum by Centrifi Chem 400 (*Yoko Nishikawa*).
Analysis of random errors in the rate assay system—Hitachi 726: Two reports (*Ikuo Motoyama*).
Automated differential determination of ketone bodies in blood (*Yutaka Harano*).
Automatic determination of 17-hydroxy corticosteroids (*Yoshirou Kawano*).
A rapid method for determination of ionized calcium in serum, using the DuPont ACA (*Katsuji Kobori*).

*Five issues of JJCLA are published in each of its annual volumes. 1982 prices are 5000 yen surface mail and 10 000 yen airmail; single copies cost 2200 and 4000 yen respectively. Orders should be sent to the Japanese Association of Clinical Laboratory Automation, 9–3 Nakamaru-cho, Itabashiku, Tokyo, Japan.*

